# Structural Basis of pppGpp Binding to the N-Terminal Domain of the Bifunctional RelA/SpoT Homolog Rel_Seq_: Crystal Structure and MD Analysis

**DOI:** 10.3390/ijms27125509

**Published:** 2026-06-18

**Authors:** Svetlana A. Korban, Zoya A. Spiridonova, Pavel S. Kasatsky, Alexey V. Shvetsov, Vladislav V. Gurzhiy, Alena Paleskava, Anna A. Kulminskaya, Andrey L. Konevega, Daria S. Vinogradova

**Affiliations:** 1 Petersburg Nuclear Physics Institute Named by B.P. Konstantinov of National Research Centre “Kurchatov Institute”, Gatchina 188300, Russiaspiridonova_za@pnpi.nrcki.ru (Z.A.S.); kasatskiy_ps@pnpi.nrcki.ru (P.S.K.); shvetsov_av@pnpi.nrcki.ru (A.V.S.); polesskova_ev@pnpi.nrcki.ru (A.P.); konevega_al@pnpi.nrcki.ru (A.L.K.); 2 Laboratory of Biomolecular NMR, St. Petersburg State University, Saint Petersburg 199034, Russia; 3 Institute of Biomedical Systems and Biotechnologies, Peter the Great St. Petersburg Polytechnic University, Saint Petersburg 195251, Russia; 4 National Research Centre “Kurchatov Institute”, Moscow 123098, Russia; 5 Crystallography Department, Institute of Earth Sciences, St. Petersburg State University, Saint Petersburg 199034, Russia

**Keywords:** Rel_Seq_, bifunctional enzyme, RelA/SpoT homolog, stringent response, alarmone, pppGpp, X-ray, crystal structure, molecular dynamics, nanoDSF

## Abstract

RelA/SpoT homologue family enzymes participate in controlling the cellular levels of the alarmone (p)ppGpp, thereby activating the stringent response and promoting survival under stress conditions. These proteins contain an N-terminal catalytic domain and a C-terminal regulatory domain. They catalyze both the synthesis of ppGpp/pppGpp from ATP and GDP/GTP and their hydrolysis to GDP/GTP and pyrophosphate. Here, we report the crystal structure of the N-terminal domain of Rel from *Streptococcus equisimilis* in complex with pppGpp at 3.2 Å resolution. The asymmetric unit contains a dimer with asymmetric ligation: pppGpp occupies only the synthetase site in one monomer, whereas in the other monomer, it is bound in both the hydrolase and synthetase sites. The two monomers exhibit distinct conformational states, with pronounced rearrangements of the flexible loops surrounding the binding pockets, including the α2/α3 and α8/α9 loops that act as steric gates. Molecular dynamics simulations support the dual binding arrangement and reveal additional probable transient binding sites, including a region in the linker between hydrolase and synthetase subdomains. These findings provide a structural framework for understanding how pppGpp binding modulates the opposing catalytic activities of bifunctional Rel enzymes and suggest possible mechanisms for (p)ppGpp-mediated autoregulation.

## 1. Introduction

Bacteria rapidly sense and adapt to environmental changes through complex strategies such as the stringent response, a pleiotropic process that broadly reshapes cellular metabolism and gene expression [1,2,3,4,5,6]. It is triggered by the intracellular accumulation of the alarmone signal molecules (p)ppGpp—guanosine 3′-diphosphate 5′-triphosphate (pppGpp) and guanosine 3′,5′-bisdiphosphate (ppGpp)—which are GTP/GDP derivatives bearing pyrophosphate groups at the ribose 3′-hydroxyl position [7].

Cellular (p)ppGpp levels are controlled by the RelA/SpoT homologue (RSH) family of proteins, as well as by small alarmone synthetases (SASs) and small alarmone hydrolases (SAHs), which exclusively catalyze (p)ppGpp synthesis and hydrolysis, respectively, in response to diverse stresses [2,3,4,5,8,9,10]. Biochemically, Rel-like synthetases primarily generate pppGpp by transferring a pyrophosphate group from ATP to GTP, while ppGpp is formed either directly from GDP or via subsequent hydrolysis of pppGpp. When GTP is in excess over GDP, the enzyme predominantly produces pppGpp, indicating that pppGpp, rather than ppGpp, is the physiologically relevant immediate reaction product [11]. Interestingly, the synthetase activity of Rel/RelA is stimulated by the alarmone itself, with pppGpp being the most potent activator [12,13,14]. Among RSH enzymes, the bifunctional, ribosome-associated “long” Rel proteins are predominant in bacteria. The Rel homologue from *Streptococcus equisimilis* (Rel_Seq_) is a representative that comprises an N-terminal catalytic domain (NTD) and a C-terminal regulatory domain (CTD) [11,15,16,17,18]. The NTD itself possesses two distinct subdomains—a hydrolase (HD) domain for (p)ppGpp degradation and a synthetase (SYN) domain for its synthesis—endowing Rel_Seq_ with both opposing catalytic activities. Notably, deletion of the CTD shifts the catalytic balance dramatically—activating synthetase activity ~12-fold while suppressing hydrolase activity ~150-fold—underscoring the critical regulatory role of the CTD in tuning the enzyme’s functional mode [8,15,18,19].

In bifunctional Rel enzymes, nucleotide binding to either the HD or the SYN domain primes the enzyme for the corresponding activity while allosterically suppressing the opposing catalytic site [18,20]. Consequently, the relative concentrations of nucleotides in solution shift the enzyme’s dynamic conformational equilibrium toward the functionally favored state, thereby preventing futile cycles of simultaneous (p)ppGpp synthesis and degradation [20].

RSH enzymes are also targeted by diverse regulatory proteins that modulate their activity. In a recent study, camelid nanobodies were used to target allosteric sites on Rel and RelA, either mimicking ribosome effects on synthetase activity or stabilizing hydrolysis-competent states [21]. Structural and biochemical analyses have shown that allosteric regulators—both endogenous proteins and camelid nanobodies—constrain the conformational landscape of long RSH enzymes, establishing allostery as a central mechanism for (p)ppGpp homeostasis and demonstrating that allosteric sites represent promising targets for antimicrobial strategies against stringent response regulators [21]. This suggests that long RSH enzymes possess allosteric hotspots that sense the bacterial metabolic state and provide crucial checkpoints to prevent (p)ppGpp overproduction [12,20].

In our recent experiments, we found that NTD of Rel_Seq_ (Rel_Seq385_) is conformationally most stable in its alarmone-bound form and exhibits a twofold-higher affinity for pppGpp than for ppGpp [22]. Given the critical role of alarmone-mediated regulation (especially by pppGpp), the extensive allostery, and the diversity of targets, we suggest that alarmone itself may shape the enzyme’s conformational and allosteric architecture. Currently, structural data remain limited yet essential because they illuminate not only the mechanism of enzyme regulation by various effectors but also the homeostatic control of alarmone levels in the cell. Specifically, we propose that preferential stabilization of the alarmone-bound state may help maintain (p)ppGpp within a range that supports proper cellular homeostasis, balancing the need for stringent-response signaling against the toxicity of alarmone overaccumulation [16,17,18]. 

Here, using an enzymatically active Rel_Seq385_, we determined its crystal structure in complex with pppGpp. This allowed us to visualize the architecture of the synthetase and hydrolase active sites and to identify key residues directly involved in pppGpp coordination. Furthermore, given that the Rel_Seq_ fragment under study is a two-domain protein with inherent flexibility, we performed molecular dynamics simulations to examine the behavior of the Rel_Seq385_·pppGpp complex in solution and to identify possible transient binding sites beyond those observed in the crystal. Thus, our work provides a structural description of pppGpp binding to the N-terminal domain of Rel_Seq_. Through molecular dynamics simulations, we reveal a potential additional binding hotspot that may be relevant for allosteric regulation. These findings extend our understanding of how pppGpp engages the catalytic domains of bifunctional RSH enzymes and offer a structural basis for further functional studies.

## 2. Results

### 2.1. Conformational Stability of Rel_Seq385_

Experiments with full-length Rel_Seq_ in vitro are often hindered by its limited solubility, as the protein displays a strong propensity to aggregate and precipitate in response to even small deviations from a narrow set of optimal buffer conditions, whereas truncation of the CTD markedly improves its solubility while preserving catalytic activity [23,24]. In our standard purification protocol, the storage buffer contained glycerol, which is commonly used to enhance protein solubility and prevent aggregation or precipitation during isolation and storage [22]. However, under crystallization conditions, the presence of glycerol reproducibly led to the formation of precipitates instead of well-ordered crystals, most likely due to interference of glycerol with crystal nucleation and growth. For preparation of Rel_Seq385_ for crystallographic analysis, we therefore modified the storage buffer by omitting glycerol from the final composition. The conformational stability and aggregation state of the protein was assessed by nanodifferential scanning fluorimetry (nanoDSF) as described previously [22], after storage at 4 °C or 25 °C for up to one week, as well as following multiple freeze–thaw cycles and storage at −80 °C. Although the absence of glycerol led to a decrease in conformational stability, as reflected by a reduction in the melting temperature by approximately 4–6 °C, the shape of the unfolding curve, baseline fluorescence, and overall transition amplitude remained unchanged, indicating preservation of the overall protein fold and confirming that the protein remained suitable for subsequent structural analysis under these conditions. Importantly, monitoring of light scattering during nanoDSF measurements revealed no detectable aggregation under any of the tested storage conditions throughout the entire observation period. During the observation period of one week, the protein retained its conformational integrity when stored at 4 °C or 25 °C, as well as upon storage at −80 °C with multiple freeze–thaw cycles using liquid nitrogen (Figure S1).

### 2.2. Rel_Seq385_ Catalytic Activities in (p)ppGpp Turnover

Before crystallization, we assessed the enzymatic activity of Rel_Seq385_ to confirm that the purified protein was catalytically competent. Structural and functional studies of RSH proteins have shown that active-site conformation and the positioning of key catalytic elements depend on the presence of substrate or product and can differ substantially between active and inactive enzyme states [25,26]. We assessed the catalytic activity of the enzyme under varying concentrations of magnesium and manganese ions, which play key roles in alarmone synthesis and hydrolysis, respectively.

#### 2.2.1. Synthesis Activity

**Figure 1:**
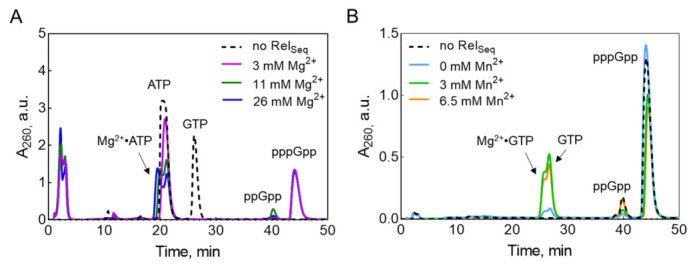
Catalytic activities of Rel_Seq385_. Chromatographic analysis of (**A**) synthetase activity of the enzyme measured at increasing Mg^2+^ concentrations. (**B**) Hydrolysis activity measured at increasing Mn^2+^ concentrations in the presence of Mg^2+^. Anion-exchange chromatography was performed with a 50–250 mM LiCl linear gradient. Additional control experiments at varying manganese concentrations are shown in Supplementary Figure S2D.

By coordinating the phosphate groups of the substrates, Mg^2+^ stabilizes their reaction competent configuration and promotes activation of the 3′-OH group of the acceptor nucleotide [27,28]. Structural and functional studies of RSH enzymes, including Rel_Seq_ and Rel*_Mtb_* from *Mycobacterium tuberculosis*, have shown that (p)ppGpp synthetase activity depends on the balance between the total nucleotide substrate concentration and Mg^2+^ levels, with the optimum approximately matching the combined concentration of nucleotide substrates [23,28]. It has been shown that Rel_Seq_ exhibits maximal pppGpp synthetase activity at 3–7 mM MgCl_2_ when the total nucleotide substrate concentration is around 4 mM, whereas increasing the total substrate concentration to 12 mM shifts the activity optimum to 10–15 mM MgCl_2_, closely matching the combined concentration of ATP and GTP. This is consistent with a model in which the enzyme acts on the Mg^2+^·ATP and Mg^2+^·GTP complexes, and it is the concentration of these Mg–nucleotide species, rather than the total ATP/GTP levels, that determines the effective rate of (p)ppGpp synthesis *in vitro*. Excess Mg^2+^ beyond this optimal range leads to a reduction in synthesis rates, likely due to subtle changes in Mg^2+^·ATP coordination and active site conformation [16,23,28]. In our experiments, Rel_Seq385_ synthesized pppGpp equally effectively in the presence of 10 mM ATP and 4 mM GTP over a range of Mg^2+^ concentrations (Figure 1A) and across different temperatures and incubation times (Figure S2B). At higher Mg^2+^ concentrations, the synthetase activity of Rel_Seq385_ in our experiments remained unchanged, indicating that the enzyme did not lose activity under these conditions and the excess Mg^2+^ was present in ATP-bound and, probably, AMP-bound forms (Figure S2C) [29,30,31].

#### 2.2.2. Hydrolysis Activity

In long Rel/SpoT-like enzymes, 3′-pyrophosphohydrolase activity is Mn^2+^-dependent. Quantitative assays with the purified N-terminal domain of Rel_Seq_ have demonstrated Mn^2+^-dependent hydrolysis of (p)ppGpp at millimolar Mn^2+^ concentrations, with no detectable activity toward (p)ppApp, indicating a clear guanosine specificity of the hydrolase reaction [23,32].

The presence of Mg^2+^ in the concentration range required for synthetase activity (5–10 mM) does not suppress Mn^2+^-dependent hydrolase activity, indicating that Mg^2+^ at these levels does not interfere with alarmone hydrolysis by the enzyme [32]. Under our experimental conditions with 14 mM Mg^2+^, the hydrolase activity of Rel_Seq385_ was strongly Mn^2+^-dependent, with only low residual activity detectable in the absence of Mn^2+^ (Figure 1B). We observed rapid hydrolysis of pre-synthesized (p)ppGpp and free GTP, including the Mg^2+^·GTP form (Figure S2D). These observations are consistent with a model in which Mg^2+^ supports the synthetase center, whereas Mn^2+^ activates the hydrolase domain of Rel_Seq_ [23,32].

Biochemical characterization of purified Rel_Seq385_ demonstrated that its catalytic activity remained high throughout all purification steps, storage, and buffer adjustments, thereby ensuring that the protein can be used for structural analysis of alarmone complexes that faithfully represent a physiologically active state of the enzyme. In addition, our experiments showed that an excess of Mg^2+^ ions did not interfere with the catalytic activity of the enzyme.

### 2.3. Stability Characterization of the Rel_Seq_∙pppGpp Complex

**Figure 2:**
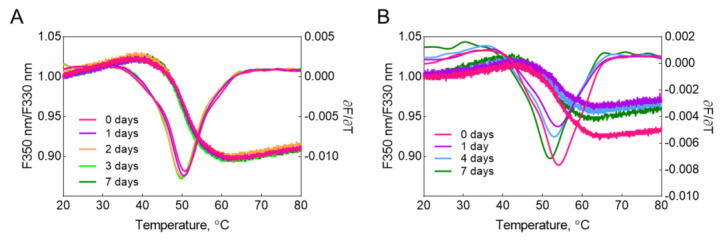
Melting curves of Rel_Seq385_ (**A**) and Rel_Seq385_·pppGpp complex (**B**) during 7 days of incubation at 25 °C.

To assess the conformational stability of the Rel_Seq385_·pppGpp complex under temperature and time conditions relevant for crystallization, we incubated the protein and its pppGpp complex at 25 °C and monitored time-dependent conformational changes by analyzing their thermal denaturation profiles (Figure 2). The conformational stability of the protein remained unchanged over the entire observation period (7 days), as evidenced by an invariant melting temperature and overall unfolding profile (Figure 2A). The Rel_Seq385_·pppGpp complex exhibited subtle but detectable changes in its thermal denaturation behavior over time (Figure 2B). The overall shape of the unfolding curve and the melting temperature of the complex remained unchanged. However, the total amplitude of the fluorescence signal change associated with the conformational transition progressively decreased, consistent with a possible reduction in the fraction of protein (or protein·alarmone complexes) undergoing a cooperative transition, including potential alterations in complex stoichiometry [22,33,34].

### 2.4. Crystallographic Structure of the Rel_Seq385_·pppGpp Complex

The structure of Rel_Seq385_ in complex with pppGpp was solved and refined at 3.2 Å resolution by the molecular replacement method using monomer A of the 1VJ7 structure as a search model. The crystal belongs to C2 space group with unit cell parameters a, b, c = 173.75, 44.98, 126.43 (Å).

**Figure 3:**
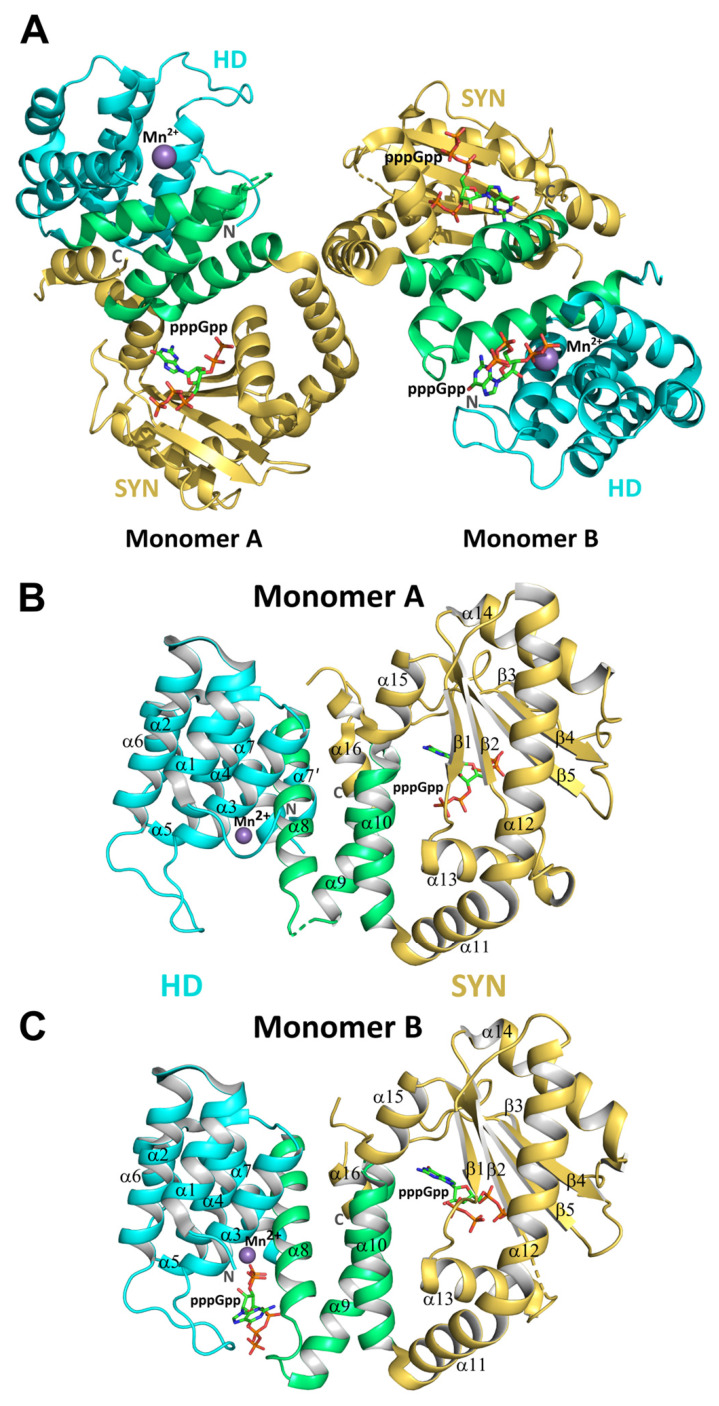
(**A**) The overall view of the Rel_Seq385_·pppGpp crystal structure in an asymmetric unit. The hydrolase domain (HD) is colored cyan, the central three-helix bundle is colored light green and the synthetase domain (SYN) is colored gold. (**B**) Monomer A in complex with Mn^2+^ bound in HD domain and pppGpp bound in SYN domain. (**C**) Monomer B in complex with Mn^2+^ in HD domain and pppGpp bound in both HD and SYN domains. pppGpp molecules (sticks) are color-coded with carbon (green), oxygen (red), nitrogen (blue), and phosphorus (orange). Mn^2+^ ion in HD site is shown as a purple sphere. For monomers A and B, the secondary structure elements of Rel_Seq385_ are shown, with α-helices (α1–α16) and β-strands (β1–β5) numbered.

The crystallographic asymmetric unit contains a Rel_Seq385_ dimer, revealing two monomers captured in distinct conformational states (Figure 3). In monomer A, the alarmone pppGpp binds exclusively to the synthetase domain, whereas monomer B exhibits pppGpp bound in both the synthetase and hydrolase domains. Analysis of the crystallographic Rel_Seq385_ dimer interface using the PDBePisa server did not predict it to be a physiologically relevant dimerization interface, suggesting that it likely arises from crystal packing.

While the protein backbone was well fitted into the electron density map, certain side chains were poorly resolved due to local weak density. Several loop regions were invisible in the density and thus omitted from modeling (monomer A: residues 113–123, 154–159, 344–360; monomer B: residues 111–130, 260–264, 342–362).

The overall structure of the Rel_Seq385_ monomer consists of the hydrolase (residues 1–159) and synthetase (residues 176–372) domains joined by an overlapping central three-helix bundle (residues 135–195).

#### 2.4.1. The pppGpp-Hydrolase Active Site of Rel_Seq385_

The hydrolase domain of Rel_Seq385_ consists of α-helices 1–7 and shows a structurally conserved fold (Figure 3B,C).

The binding pocket for the substrate (p)ppGpp is surrounded by the extended α2/α3 loop (residues 40–50) that connects helices α2 and α3, helix α3, the conserved “HD” motif between helices α4 and α5 (residues His77, Asp78), and helix α8 of the central three-helix bundle.

**Figure 4:**
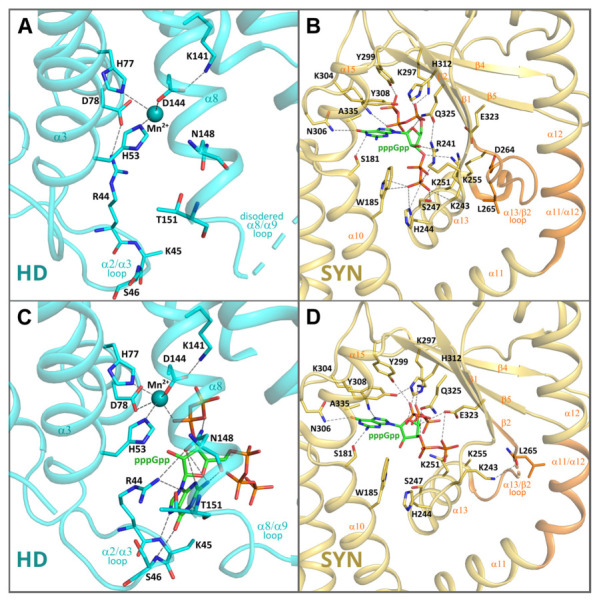
Active site architecture and pppGpp binding in the HD (cyan) and SYN (gold) domains of the bifunctional enzyme Rel_Seq385_. In monomer A, the HD active site is unoccupied (**A**), whereas the SYN domain harbors a bound pppGpp molecule (**B**). In monomer B, pppGpp is present in both HD (**C**) and SYN (**D**) active sites. HD and SYN domains of the two monomers of Rel_Seq385_ are shown and colored in cyan and gold, respectively. The alarmone pppGpp is depicted as a stick model with atoms color-coded: carbon (green), oxygen (red), nitrogen (blue), and phosphorus (orange). Secondary structure elements are labeled in cyan (HD domain) and orange (SYN domain). Interatomic contacts are shown as gray dashed lines. The corresponding 2mFo-DFc electron density maps (contoured at 1σ) for pppGpp in the HD and SYN domains of both monomers are provided in the Supplementary Material (Figure S5).

**Figure 5:**
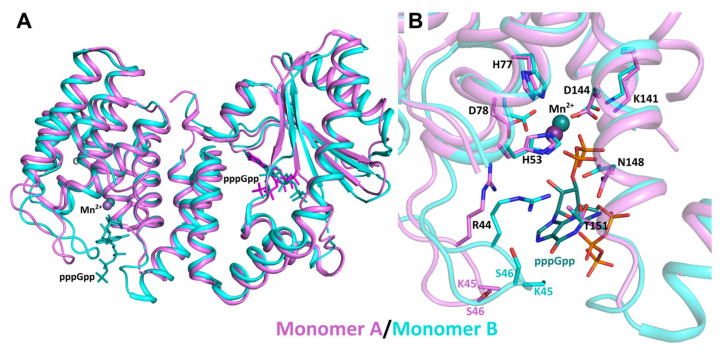
Structural comparison of the two Rel_Seq385_ monomers. (**A**) Cartoon representation of superimposed monomer A (violet) and monomer B (cyan) structures. pppGpp molecules are shown as sticks colored according to the monomer color. (**B**) Structural alignment of the HD active sites of monomer A (violet) and monomer B (cyan), showing key catalytic residues as sticks. pppGpp bound in the HD site of monomer B is shown as sticks. Mn^2+^ ions are shown and colored according to the monomer color.

In the unoccupied HD pocket of monomer A, a divalent metal ion (Mn^2+^) is coordinated by the side chain atoms of His53, His77, and Asp144 (Figure 4A). As observed in monomer A, the α8/α9 loop fragment (residues 154–159) is disordered in the electron density maps in the ligand-free state. Upon pppGpp binding, the flexible loops surrounding the binding pocket undergo pronounced conformational changes (Figure 4C and Figure 5). Specifically, in monomer B the α8/α9 loop becomes ordered, accompanied by coordinated movements of the α2/α3 and α8/α9 loops, which together form a cap beneath the entrance to the binding cleft, acting as steric gates. The α2/α3 loop is displaced and shifts proximally toward the substrate-binding pocket. This repositioning leads to local displacements of residues Arg44, Lys45 and Ser46. As a result, Arg44 loses its salt bridge with Asp78 and instead forms hydrogen bonds with the side chain atoms of Asn148 and interacts with the guanine base of pppGpp, while Lys45 and Ser46 also engage in interactions with the alarmone. This disruption of the salt bridge releases Asp78, allowing it to approach the Mn^2+^ ion. Previous mutagenesis studies have demonstrated that the R44Q and D78A mutations abolish hydrolase activity of the enzyme [16], underscoring the essential role of these residues in catalysis. Consistent with the proposed role of Arg44 and Asp78 in coordinating Mn^2+^ to the 3′-diphosphate of (p)ppGpp [16], the observed rearrangement of Arg44 upon ligand binding underscores its dual role: facilitating substrate binding and contributing to the proper coordination of the metal ion essential for hydrolase activity. Despite the resolution limit of 3.2 Å and the fact that some side chains are associated with weak electron density, most residues in the active sites of the hydrolase and synthetase domains are rather well defined, allowing for reliable interpretation of the observed structural rearrangements. The conventional 2mFo-DFc maps for the HD and SYN sites are shown in Figures S3A,B and S4A,B. To reliably interpret the paired side chains of Arg44 and Asp78—especially given the weak density for Arg44—we additionally generated polder OMIT maps using phenix.polder, a tool implemented in the PHENIX software package (version 1.19.2_4158) [35]. This approach is particularly useful for verifying weak or ambiguous side-chain density. The resulting polder maps confirmed the conformations of Arg44 and Asp78 in both monomers (Figure S3C,D), thus validating our structural interpretation of their paired arrangement.

The guanine base of pppGpp is coordinated within the HD active center through hydrogen-bond interactions involving the main-chain amides of Lys45 (to N-7 of pppGpp) and Ser46 (to O-6), as well as the main-chain carbonyl of Thr151 (to N-1) and Asn148 (to N-2). Notably, the side chain of Lys45 was not resolved in the electron density and was therefore omitted from the model (atoms CG, CD, CE, and NZ in monomer A and atoms CD, CE, and NZ in monomer B). Additional stabilization of pppGpp is provided by interactions with the side chains of Arg44 and Asn148.

Turning to the phosphate moieties, the α-phosphate of the 3′-diphosphate group points Asn148 side chain, as well as with the essential manganese ion cofactor Mn^2+^. In contrast, the β-phosphate is oriented toward the solvent and does not exhibit strong restraining interactions with nearby atoms. Finally, the 5′-triphosphate group of pppGpp is exposed to the bulk solvent.

#### 2.4.2. The pppGpp-Synthetase Active Site of Rel_Seq385_

The C-terminal synthetase domain (residues 176–371) consists of 5-stranded mixed β-sheet surrounded by five α-helices (α11−α15) in a sandwich-like arrangement, which forms a solvent accessible cavity (Figure 3B,C and Figure 4B,D).

Interestingly, the SYN domains of both monomers in the asymmetric unit are occupied by pppGpp. However, the binding mode of the alarmone differs slightly between monomer A and monomer B. While the guanine moiety adopts a conserved position and forms hydrogen bonds with Ser181, Asn306, and Ala335 in both monomers, the overall conformation of pppGpp and its local interactions are not identical. In particular, the side chain of Lys304 adopts different orientations in the two monomers: in monomer A it interacts with the 5′-triphosphate group of pppGpp (Figure 4B), whereas in monomer B it contacts the guanine ring (Figure 4D).

The 3′-diphosphate and 5′-triphosphate groups exhibit distinct spatial arrangements in the two monomers, leading to a somewhat different pattern of hydrogen-bonding interactions with the same set of surrounding residues, including Trp185, Arg241, Lys243, His244, Ser247, Lys251, Lys297, Tyr299, His312, and Gln325, which are involved in binding and stabilizing pppGpp.

Beyond these differences in the bound alarmone’s orientation, the two monomers exhibit pronounced conformational heterogeneity in the surrounding structural elements. In monomer A, the catalytic loop α13/β2 (residues 254–266) is stabilized and ordered through van der Waals interactions with the side chains of the helical fragment comprising residues 209–217 (α11/α12) (Figure 4B). Conversely, in monomer B the α11/α12 fragment shows partial disorder, as evidenced by poorly defined side-chain electron density. This loss of rigidity weakens the interactions with the catalytic loop, thereby destabilizing the α13/β2 loop (Figure 4D). Thus, while in monomer A the catalytically essential residue Asp264 is clearly visible in the electron density and forms a salt bridge with the side chain of Lys255, in monomer B this interaction is lost due to disorder of the α13/β2 loop, which renders these residues poorly defined in the density. Consequently, the side chain of Lys255 is not resolved, and Asp264 is not visible at all.

Hogg and colleagues identified Asp264 and Glu323 as the catalytically essential carboxylate residues in the synthetase active site, a finding supported by mutagenesis: the D264G and E323Q mutations eliminate detectable synthetase activity [16]. They proposed that Glu323 coordinates Mg^2+^ and serves as the GTP/GDP 3′-OH proton acceptor during the (p)ppGpp-synthetase reaction. In our structure, however, the side chain of Glu323 in monomer A exhibits fragmented electron density, suggesting mobility and a tendency to form ionic interactions with the side chains of Lys243 or Lys251. In monomer B, by contrast, Glu323 becomes more ordered and appears to form the interactions with the bound pppGpp. Furthermore, Lys243, which was earlier implicated as a probable ATP-phosphate binding residue [16], in monomer B undergoes a concomitant rearrangement away from the active site to interact with the backbone carbonyl of Leu265. We additionally generated polder OMIT maps [35] for Lys243 and Glu323, which confirmed the mobility of Glu323 in monomer A and its ordered conformation in monomer B, as well as the repositioning of Lys243 away from the active site (Figure S4C,D).

Taken together, our data suggest that the conformational state of the synthetase active site depends on the integrity of a structural framework that coordinates coupled and concerted movements of the loops surrounding the binding pocket.

### 2.5. Molecular Dynamics Simulations of Rel_Seq385_·pppGpp

RSH enzymes are increasingly recognized as conformationally dynamic regulators with relative domain rearrangements and flexible linkers playing a key role in modulating (p)ppGpp metabolism. Structural and bioinformatic studies of RSH proteins have highlighted that this plasticity is an intrinsic feature of the superfamily and is closely linked to alarmone binding and allosteric control [17,26,36,37]. Rel_Seq385_ conforms to this paradigm: despite being a truncated construct, it comprises two domains connected by a flexible linker. While X-ray crystallography provides a static snapshot of the protein, it cannot capture the full range of motions or the possible existence of additional binding sites that may be sampled in solution. Previous computational studies on (p)ppGpp–protein systems have suggested that such transient sites can be functionally important [15,27]. Therefore, to characterize the conformational landscape of Rel_Seq385_ in solution, to search for potential alarmone binding hotspots beyond those observed in the crystal, and to independently validate our structural model, we performed molecular dynamics (MD) simulations of the Rel_Seq385_·pppGpp complex.

Throughout the simulations, Rel_Seq385_ largely preserved its secondary structure and overall fold, whereas the magnitude and temporal pattern of backbone root mean square deviation (RMSD) pointed to pronounced interdomain motions within the NTD, consistent with the intrinsic flexibility described for Rel/RelA N-terminal domains [17,26].

**Figure 6:**
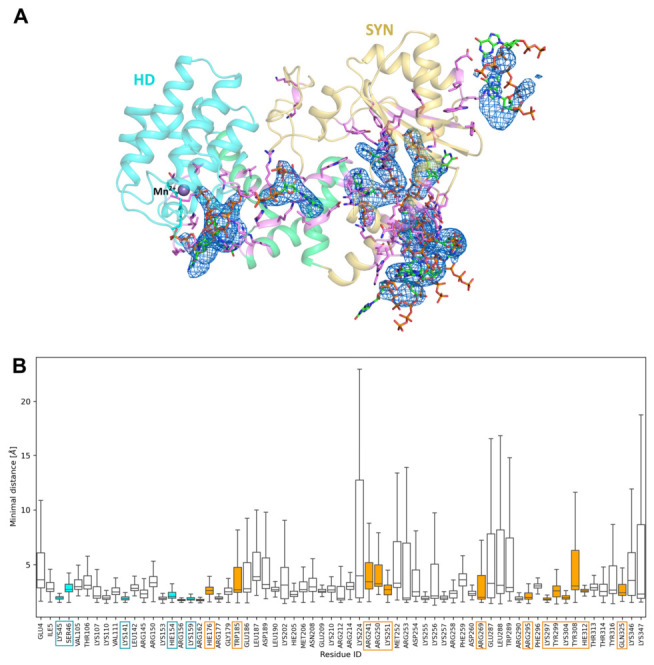
MD simulations of Rel_Seq385_ with 32 molecules of pppGpp in periodic water box with dimensions of 90 × 90 × 90A. (**A**) Probability maps for pppGpp positions shown on the central structure of the most probable Rel_Seq385_ conformation based on conformational clustering. Domain coloring follows Figure 3. The probability maps are visualized as blue meshes. Possible pppGpp positions in the predicted binding zones are shown as sticks, color-coded with carbon (green), oxygen (red), nitrogen (blue), and phosphorus (orange). Residues with a median minimal contact distance ≤4 Å are shown as violet sticks. The Mn^2+^ ion in the hydrolase domain is shown as a purple sphere. (**B**) Box plot showing minimal distances between pppGpp and each protein residue (only residues with median minimal distance below 4 Å are displayed) observed during MD simulations. Residues whose contacts are consistent with the crystallographic binding sites are highlighted: the corresponding boxes are colored cyan (HD site) or orange (SYN site), and the residue labels on the x-axis are enclosed in boxes of the same colors.

To identify preferred alarmone interaction regions, we extracted pppGpp–protein contacts along the trajectories by calculating pairwise distances between the ligand and protein residues and generated probability-weighted contact maps. For each monomer in the crystallographic dimer, residue-wise contact frequencies with pppGpp were calculated, highlighting hotspots of recurrent interaction. A contact between pppGpp and a given protein residue was considered stable if the median minimal distance between any atoms of the two entities was ≤4.0 Å over the course of a simulation run. To assess the reproducibility of the simulations, we performed two independent MD runs (replicas), differing only in the initial atomic velocities assigned from the Maxwell-Boltzmann distribution by varying the random seed (gen-seed). Analysis of all trajectories revealed no substantial differences in the overall contact patterns between the protein and the alarmone; the identified residue-wise contact frequencies and the localization of high-probability binding regions were consistent across replicas, confirming that the observed ligand-binding hotspots are robust and not an artefact of a particular random initial velocity assignment. In monomer B, this analysis revealed several high probability patches, including positions that spatially overlapped with the pppGpp binding sites observed in our crystal structure, thereby supporting the functional relevance of the crystallographic binding sites (Figure 6).

In addition to the hydrolase and synthetase sites, however, the MD simulations also revealed additional zones of probable pppGpp binding. One notable such patch was localized in the linker connecting the HD and SYN domains, encompassing segments of α-helices α9 and α10. This region has previously been shown to act as a transmission core that mediates coupling between the SYN and HD domains [18]. Furthermore, an allosteric (p)ppGpp binding site was identified in *E. coli* RelA at the interface between the SYN and pseudo-HD domains where pppGpp binding promotes SYN activity by antagonistic allosteric cross-talk between the two NTD domains [12]. In our MD simulations, we observed a similarly located hotspot in Rel_Seq385_, comprising residues Arg150, Lys153, His154, Glu186, Leu187, Asp189, and Leu190 (Figure S6), suggesting that a comparable allosteric mechanism may also operate in this enzyme. Additionally, MD identified binding areas included a region within α-helix α11 and the loop following α-helix α14 (residues 287–290).

MD simulations of monomer A showed a similar binding pattern for the HD and SYN domains but did not reveal linker occupancy. This may reflect differences in the starting conformations of the two monomers, as well as the transient nature of alarmone binding in the linker.

## 3. Discussion

Rel from *S. equisimilis* is a bifunctional long RSH enzyme that catalyzes both (p)ppGpp synthesis and hydrolysis, playing a central role in stringent response. In this work, we focus on the N-terminal 1–385 Rel fragment (Rel_Seq385_), which corresponds to the catalytic core harboring hydrolase and synthetase subdomains. Our biochemical assays confirmed that Rel_Seq385_ exhibits robust catalytic activities: (p)ppGpp synthesis via ATP–GTP/GDP 3′-pyrophosphotransferase and Mn^2+^-dependent 3′-pyrophosphohydrolase activity. Although our recent study demonstrated enhanced conformational stability of Rel_Seq385_ upon alarmone binding [22], structural information on how (p)ppGpp engages the catalytic domains remains limited. We now report its crystal structure in complex with pppGpp.

Our Rel_Seq385_·pppGpp structure (PDB 24IO) reveals remarkable similarities to the previously reported Rel_Seq_·GDP complex (PDB 1VJ7), in which the asymmetric unit likewise contains two monomers with distinct domain orientations and ligation states [16]. In the Rel_Seq_·GDP structure, monomer 1 (the equivalent of our monomer A) has GDP bound only in its synthetase domain, while monomer 2 (the equivalent of our monomer B) contains GDP in its synthetase domain and additionally harbors an unusual GDP derivative, ppG2′:3′p, in its hydrolase domain. A similar pattern is observed in our Rel_Seq385_·pppGpp structure: monomer A contains pppGpp exclusively in the synthetase domain, whereas monomer B has pppGpp bound in both the hydrolase and synthetase domains (structural superposition is shown in Figure S7). Despite the different ligands, key catalytic residues in the corresponding binding pockets exhibit similar orientations, underscoring the structural integrity of the active sites. This dual binding mode is further supported by a *Staphylococcus aureus* Rel (Rel_Sa_) structure in complex with pppGpp (PDB 7OIW, chain B, article not published). Both Rel_Seq_ and Rel_Sa_ share a conserved long-RSH architecture with N-terminal catalytic domain and a C-terminal regulatory region. Rel_Sa_ is essential for stringent response initiation under amino acid limitation and stress conditions, and loss of its hydrolase activity (e.g., in clinical Rel mutants) leads to (p)ppGpp overaccumulation, attenuated cell growth, and altered antibiotic tolerance [38,39]. The Rel_Sa_ structure shows a similar binding pattern, with pppGpp bound to both the HD and SYN domains simultaneously. Structural superposition revealed that the hydrolase domain of Rel_Seq385_ monomer B and that of Rel_Sa_ are nearly identical, exhibiting very similar coordination of the pppGpp molecule and identical positions of the catalytic residues (Figure S9A,B). In contrast, superposition of the synthetase domains showed that the conformation of Rel_Sa_ SYN more closely resembles that of monomer A of Rel_Seq385_ than that of monomer B (Cα RMSD over the SYN domain are 1.04 Å and 1.41 Å, respectively) (Figure S8A,C). The alarmone binding mode in the synthetase pocket is virtually identical between Rel_Seq385_ and Rel_Sa_, except for the position of the 5′-triphosphate group of pppGpp, which is exposed to the solvent, explaining the observed flexibility of this tail and the corresponding minor deviations in coordinates. Together, these data indicate that pppGpp can occupy both catalytic sites in a subset of Rel proteins.

Rel_Seq385_ enzyme may exhibit conformational flexibility, as its N-terminal catalytic core consists of two subdomains (HD and SYN) connected by a mobile linker region. To explore the binding profile of pppGpp within this conformational landscape, we performed MD simulations. The MD simulations not only confirmed the dual occupancy pattern observed in monomer B but also revealed additional binding hotspots. Among these, the linker region connecting the HD and SYN domains warranted particular attention. It is known that (p)ppGpp can occupy allosteric sites between catalytic domains to mediate antagonistic coupling and conformational switching in RSH enzymes [12,13]. For instance, in *Bacillus subtilis* Rel, binding of pppGpp to such a site enhances synthetase activity [40]. Importantly, a recent study has extended these *in vitro* findings by demonstrating that allostery plays a critical physiological role, directly affecting (p)ppGpp abundance and the ability of bacteria to recover from stress [41]. 

Although we did not observe alarmone binding in the linker region or elsewhere in our crystal structure, this discrepancy likely reflects the different conditions captured by the two methods: crystallography provides a static view under packing constraints, whereas MD simulates a solvated dynamic ensemble. Thus, (p)ppGpp-binding in the linker region may be transient or dependent on a conformational state not sufficiently populated in the crystal lattice. Overall, MD captures an ensemble of accessible states, whereas crystallography provides a static snapshot of a single structure. This highlights that Rel_Seq385_ does not adopt a single rigid conformation but rather samples multiple dynamic states modulated by ligand occupancy and metal coordination. This view of conformational flexibility as a determinant of catalytic output is consistent with broader structural insights into the RSH enzyme family, in which nucleotide-driven conformational switching has emerged as a key regulatory mechanism. For example, in Rel from *Thermus thermophilus* (Rel*Tt*), GDP binding to the synthetase domain stabilizes an open, synthetase-active state, whereas ppGpp binding to the HD domain induces a closed, hydrolase-active conformation that prevents futile catalytic cycles [18].

Our MD-derived evidence for a high probability of alarmone binding in the linker region of Rel_Seq385_ deserves separate attention. This finding, together with the observation that Rel_Seq385_ binds pppGpp with higher affinity than ppGpp [22]—similar to what has been shown for RelA [12]—suggests that this interaction may represent an additional allosteric regulatory mechanism. Such a mechanism would be analogous to the product-dependent positive feedback already described for long RSH enzymes of the RelA/Rel lineage [12]. Consistent with this view, pppGpp acts as a more potent activator than ppGpp in RelA by binding to an allosteric site at the SYN–HD interface, stabilizing an open, catalytically competent conformation that relieves intra-NTD inhibition and enhances alarmone synthesis [12,13,14,42].

In summary, our data show that pppGpp can occupy both catalytic sites of Rel_Seq385_, as well as additional sites, including the linker region—a potential binding hotspot according to MD simulations. The pronounced conformational flexibility of the enzyme, together with the possible existence of transient binding sites, points to a complex regulatory landscape. Such binding patterns might help stabilize ordered conformations and contribute to buffering local alarmone levels, though further work is needed to clarify the allosteric coupling between the domains. The precise molecular mechanisms underlying these observations, as well as their full physiological relevance, await further experimental validation. Nevertheless, the structural and mechanistic insights obtained here provide a foundation for future investigations into the regulation of bifunctional RSH enzymes. Given the central role of the Rel/SpoT family in bacterial stress adaptation and the absence of close mammalian homologs, these proteins remain attractive targets for structure-guided antibacterial development.

## 4. Materials and Methods

### 4.1. Protein Expression and Purification

Protein expression and purification were performed essentially as described previously [43], with minor modifications detailed below. The N-terminal domain of Rel_Seq_ (residues 1–385, Rel_Seq385_) bearing a C-terminal hexahistidine tag was produced in *Escherichia coli* BL21 (DE3) carrying a pET21-based expression plasmid and purified as described above. Transformed cells were grown in LB medium with 100 μg/mL ampicillin at 37 °C to OD_600_ ≈ 0.6, then induced with 1 mM IPTG for 3 h at 37 °C. Cells were harvested by centrifugation at 6000 rpm for 20 min (JLA 8.1 rotor, Beckman Coulter, Inc., Brea, CA, USA) and resuspended in lysis buffer (20 mM Tris-HCl pH 7.9, 300 mM KCl, 5 mM MgCl_2_, 20% (*v*/*v*) glycerol, 10 mM imidazole, 5 mM β-mercaptoethanol, 280 μg/mL lysozyme, 0.1 mg/mL DNase I (Sigma-Aldrich, St. Louis, MO, USA), and one protease inhibitor cocktail tablet (Roche, Basel, Switzerland) per 50 mL). Cell disruption was carried out using an EmulsiFlex C3 homogenizer (Avestin, Ottawa, ON, Canada), and insoluble material was removed by centrifugation at 45,000 rpm for 30 min (Ti50.2 rotor, Beckman Coulter, Inc., Brea, CA, USA).

The clarified lysate was loaded onto a 5 mL HisTrap FF column (GE Healthcare, Chicago, IL, USA) equilibrated with buffer HT (20 mM Tris-HCl pH 7.9, 5 mM MgCl_2_, 20% (*v*/*v*) glycerol, 5 mM β-mercaptoethanol) containing 300 mM KCl and 10 mM imidazole. Rel_Seq385_ was eluted by a stepwise increase in imidazole concentration from 10 mM to 200 mM, and the pooled fractions were diluted with buffer HT containing 10 mM KCl to adjust the final KCl concentration to 50 mM. For further purification, the sample was applied to a 5 mL HiTrap Q anion-exchange column (GE Healthcare, Chicago, IL, USA) pre-equilibrated in 20 mM Tris-HCl pH 9.5, 50 mM KCl, 5 mM MgCl_2_, 20% (*v*/*v*) glycerol, and 5 mM β-mercaptoethanol. Bound protein was eluted using a linear KCl gradient from 50 mM to 1.5 M. Fractions containing Rel_Seq385_, as assessed by 10% SDS-PAGE (Figure S10A), were pooled, aliquoted, and flash-frozen in liquid nitrogen.

### 4.2. Conformational Stability of Rel_Seq385_ and Rel_Seq385_·pppGpp Complexes by nanoDSF

Conformational stability of Rel_Seq385_ was assessed essentially as described in [22]. In all experiments, samples were subjected to a temperature ramp from 20 °C to 95 °C at a heating rate of 1 °C/min. Protein stability was evaluated when stored in a buffer A of 20 mM Tris-HCl, pH 8.0, 1 M NaCl, 5 mM MgCl_2_, 1 mM DTT without glycerol and in the same buffer with 20% glycerol. The protein concentration in both cases was 130 μM. The protein was kept at 4 °C, 25 °C, and −80 °C with three freeze–thaw cycles. Conformational stability of the protein and its complex with 2 mM pppGpp at 25 °C was monitored over one week by sampling at multiple time points (0 h, 1, 2, 3, 4, 7 days). Samples (130 μM) were analyzed in storage buffer A without glycerol.

For all measurements, reaction mixtures were loaded into glass capillaries (NanoTemper Technologies GmbH, Munich, Germany) with a volume of 10 μL per capillary, and nanoDSF experiments were conducted on a Prometheus NT.48 instrument (NanoTemper Technologies GmbH, Munich, Germany). Thermal unfolding and aggregation profiles were recorded, and melting temperatures were extracted using the PR.ThermControl software package v2.0.2 (NanoTemper Technologies GmbH, Munich, Germany). Subsequent data analysis and visualization were performed in GraphPad Prism v.8.0.1 (GraphPad Software, San Diego, CA, USA).

### 4.3. Synthesis Activity of Rel_Seq385_

Alarmone nucleotide synthesis was performed essentially as described previously [43], with minor modifications. Reactions for pppGpp used buffer B (30 mM Tris-HCl pH 8.0, 100 mM NaCl) containing 10 mM ATP, 4 mM GTP, 50 μM Rel_Seq385_, and MgCl_2_ at concentrations yielding final [Mg^2+^] of 1, 3, 5, 11, or 26 mM. The mixture was incubated for 40 min at 37 °C and was applied to a Mono Q 5/50 GL (1 mL) anion exchange column (GE Healthcare, Chicago, IL, USA), pppGpp was eluted using a linear gradient of LiCl from 50 mM to 300 mM in buffer C (25 mM Tris-HCl pH 8.0, 0.5 mM EDTA).

For preparative synthesis of both pppGpp and ppGpp, reactions were carried out under the same conditions using 10 mM MgCl_2_, with ppGpp produced by substituting GDP for GTP as the guanine nucleotide substrate. Fractions containing the desired alarmone species were pooled and precipitated by addition of 1.5 M LiCl and two volumes of absolute ethanol, followed by centrifugation at 16,100× *g* for 20 min. The pellet was resuspended in water, and nucleotide concentration was determined by UV at 253 nm using a molar extinction coefficient of 13,700 M^−1^ cm^−1^. The sample was aliquoted and stored at −20 °C until use.

### 4.4. Hydrolysis Activity of Rel_Seq385_

Analytical hydrolysis of pppGpp was performed essentially as described in [32], with minor modifications. Reactions contained 5 mM pppGpp, 50 mM Tris-HCl pH 8.0, 250 mM NaCl, 14 mM MgCl_2_, Rel_Seq385_ at 22.7 μM, and MnCl_2_ at final concentrations of 0, 1, 3, or 6.5 mM. Mixtures were incubated for 40 min at 37 °C and applied to a Mono Q 5/50 GL (1 mL) anion-exchange column (GE Healthcare, Chicago, IL, USA). (p)ppGpp species were eluted using a linear gradient of LiCl from 50 mM to 300 mM in buffer C.

### 4.5. Protein Crystallization, Data Collection and Structure Determination

For crystallographic experiments, the protein Rel_Seq385_ was dialyzed against 20 mM Tris-HCl, pH 7.9, 650 mM NaCl, 2.5 mM MgCl_2_, 1 mM DTT and concentrated to 6 mg/ml using 10 kDa cut off concentrator (Merck KGaA, Darmstadt, Germany). The protein sample was then supplemented with 2 mM pppGpp, and this mixture was used for crystallization experiments. Crystals were grown at 298 K using the hanging-drop method with a 1:1 volume ratio of protein and precipitant solution containing 0.1 M Tris-HCl, pH 8.5, 24% polyethylene glycol 8000 and 1 M NaCl. The resulting crystals (Figure S10B) were harvested and immersed in the mother liquor supplemented with 20% glycerol as a cryoprotectant before being flash-frozen in liquid nitrogen. The best crystal diffracted to a resolution of 3 Å. Diffraction data were collected at 100 K using XtaLAB Synergy-S diffractometer equipped with PhotonJet-S Cu microfocus X-ray source and HyPix-6000HE detector (Rigaku Oxford Diffraction, Abingdon, UK) at the Centre for X-ray Diffraction (XRD) Studies of St. Petersburg State University. Indexing and integration were performed using autoPROC (version 20250717, Global Phasing Limited, Cambridge, UK) [44]. The high resolution of the data was truncated to 3.2 Å to improve data quality metrics. For estimate anisotropic correction, scaling was completed using STARANISO web server (http://staraniso.globalphasing.org/cgi-bin/staraniso.cgi (accessed on 13 June 2026), Global Phasing Limited, Cambridge, UK) [45]. Elliptically truncated data were used for molecular replacement with phenix.phaser (PHENIX, version 1.19.2_4158, Berkeley, CA, USA) [46].

The structure was solved by molecular replacement using the N-terminal domain of Rel/Spo homolog from *S. equisimilis* as a search model (PDB 1VJ7, monomer A) [16].

Iterative refinement, model building, and validation were performed using phenix.refine (PHENIX, version 1.19.2_4158, Berkeley, CA, USA) and Coot (version 0.9.8.96) [47,48]. Data collection and refinement statistics are presented in Table S1. The final structure has been deposited in the Protein Data Bank with the accession code 24IO.

Protein structure visualizations were prepared using PyMOL (The PyMOL Molecular Graphics System, version 2.5, Schrödinger, LLC, New York, NY, USA).

### 4.6. Bioinformatics

Interface areas were calculated using the PDBePISA server (https://www.ebi.ac.uk/pdbe/pisa/ (accessed on 13 June 2026), EMBL-EBI, Hinxton, UK) [49]. Structural homologs in the Protein Data Bank (PDB) were determined using PDBeFold (https://www.ebi.ac.uk/msd-srv/ssm/ (accessed on 13 June 2026), EMBL-EBI, Hinxton, UK) [50]. BLASTP (version 2.17.0+, NCBI, Bethesda, MD, USA) was used to compare the protein sequence against sequences of homologous proteins with known structures in the PDB [51].

### 4.7. MD Simulations

Since the solved crystal structure has missing residues, we built them using MODELLER 10.3 (UCSF, San Francisco, CA, USA) [52]. GROMACS compatible topology for pppGpp was prepared using acpype (University of Cambridge, Cambridge, UK) [53] and antechamber (UCSF, San Francisco, CA, USA) [54,55]. We built systems consisting of one Rel_Seq385_ monomer and 32 pppGpp molecules placed in random positions inside 90 × 90 × 90 Å periodic box. MD simulations were performed using GROMACS 2026 (Uppsala University/KTH, Stockholm, Sweden) with the AMBER19sb (UCSF, San Francisco, CA, USA) [56] force field and the OPC3 [57] water model. The system was neutralized by adding 100 mM NaCl to achieve zero net charge, and then energy minimized. Equilibration was performed in two stages: first, positional restraints (posres) were applied to all heavy atoms of the protein, keeping them at their initial positions, and particle velocities were assigned from the Maxwell–Boltzmann distribution at 310 K; the system was equilibrated for 5 ns using a 2 fs integration step and the V-rescale [58] thermostat (coupling time 1 ps) and C-rescale [59] barostat (coupling time 1 ps). In the second stage, restraints were removed and the system was equilibrated for 10 ns using the Nosé–Hoover thermostat [60,61] (coupling time 2 ps) and Parinello–Rahman barostat [62]. The final equilibrated state was used for 250 ns of MD simulations with the same parameters as in the second stage.

## Data Availability

Coordinates and structure factors of Rel_Seq385_ have been deposited in the PDB under accession code 24IO: https://www.rcsb.org/structure/24IO (accessed on 18 March 2026). X-ray diffraction dataset is available in the Zenodo repository at https://zenodo.org/records/18876971 (accessed on 5 March 2026).

## Supplementary Materials

The following supporting information can be downloaded at: https://www.mdpi.com/article/10.3390/ijms27125509/s1.
