# Supplementary material for: Structural Basis of pppGpp Binding to the N-Terminal Domain of the Bifunctional RelA/SpoT Homolog RelSeq: Crystal Structure and MD Analysis
Source: Int J Mol Sci. 2026 Jun 18;27(12):5509. doi: 10.3390/ijms27125509 (PMC13299517; doi:10.3390/ijms27125509)
Supplement: Supplementary file 1 [file ijms-27-05509-s001.zip › ijms-4323146-supplementary.pdf]

SUPPLEMENTARY MATERIAL

Structural Basis of pppGpp Binding to the N-Terminal Domain of the Bifunctional RelA/SpoT Homolog Rel_{Seq}: Crystal Structure and MD Analysis

Svetlana A. Korban[1,2], Zoya A. Spiridonova[1], Pavel S. Kasatsky[1], Alexey V. Shvetsov[1,3,4], Vladislav V. Gurzhiy[5], Alena Paleskava[1,3], Anna A. Kulminskaya[1,4], Andrey L. Konevega[1,3,4], Daria S. Vinogradova[1*]

[1] Petersburg Nuclear Physics Institute Named by B.P. Konstantinov of National Research Centre "Kurchatov Institute", Gatchina 188300, Russia

[2] Laboratory of Biomolecular NMR, St. Petersburg State University, Saint Petersburg 199034, Russia

[3] Institute of Biomedical Systems and Biotechnologies, Peter the Great St. Petersburg Polytechnic University, Saint Petersburg, 195251, Russia

[4] National Research Centre "Kurchatov Institute", Moscow 123098, Russia

[5] Crystallography Department, Institute of Earth Sciences, St. Petersburg State University, Saint Petersburg 199034, Russia

*Correspondence author: vinogradova_ds@pnpi.nrcki.ru

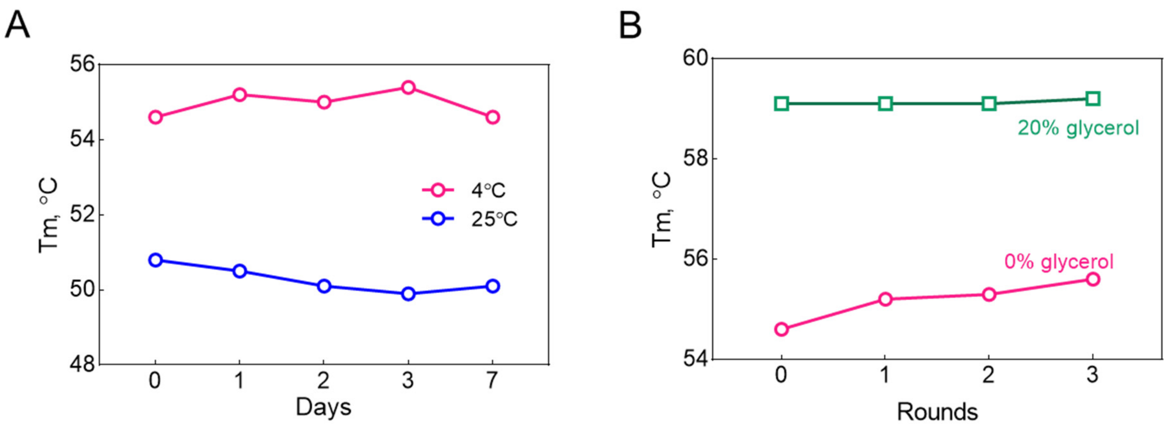

Figure S1. Thermal stability analysis of Rel_{Seq385} protein. **(A)** Melting temperatures (Tm) of Rel_{Seq385} upon incubation of the protein at 4 °C or 25 °C. **(B)** Melting temperatures (Tm) of Rel_{Seq385} (stored at -80°C) under different glycerol concentrations in storage buffer, during repeated freeze-thaw cycles in liquid nitrogen.

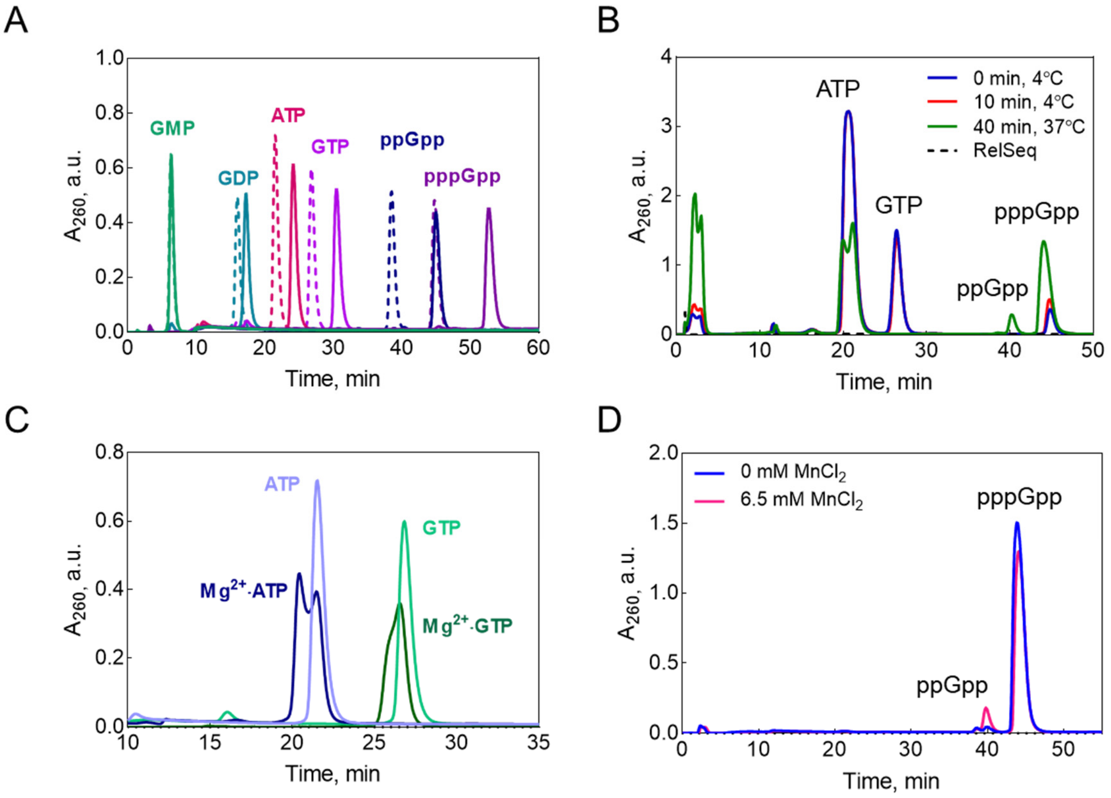

Figure S2. Chromatograms of nucleotides and alarmones. **(A)** Anion-exchange chromatography of ATP, GMP, GDP, GTP and the alarmones (p)ppGpp using a 50–250 mM (solid line) or 50–300 mM (dash line) LiCl linear gradient. **(B)** Analysis of Rel_{Seq385} synthetase activity as a function of incubation conditions. pppGpp synthesis was performed with 10 mM ATP and 4 mM GTP at 4°C for 0 min (blue line), 10 min (red line),

or at 37°C for 40 min (green line). Anion-exchange chromatography of products performed using a 50–300 mM LiCl linear gradient. **(C)** Anion-exchange chromatography of ATP, GTP and their magnesium forms performed using a 50–300 mM LiCl linear gradient. **(D)** Hydrolysis of pppGpp as a function of manganese ion concentration performed using a 50–300 mM LiCl linear gradient.

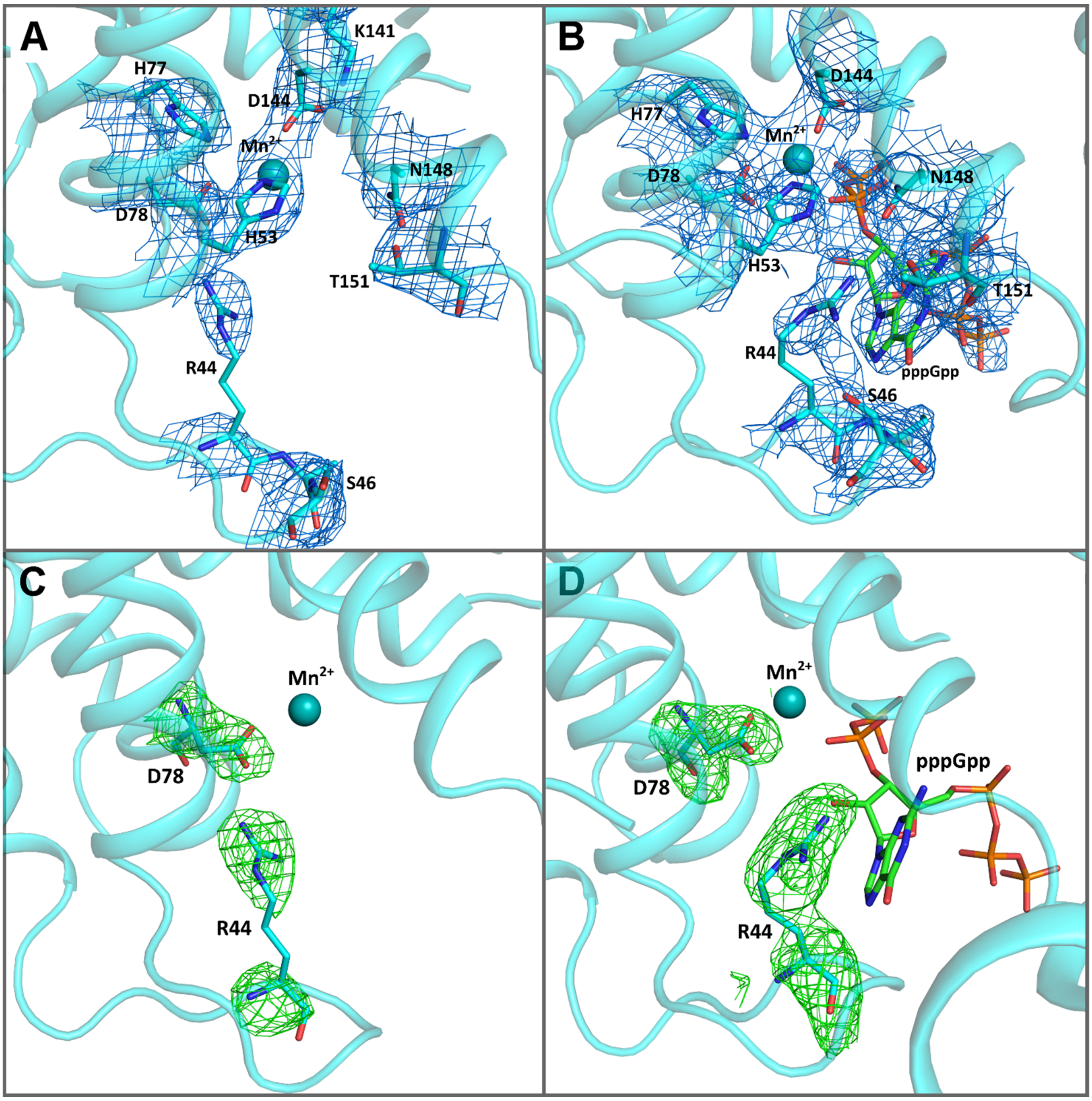

Figure S3. Electron density maps for the Rel$_{Seq385}$ hydrolase active site. **(A, B)** 2mFo-DFc maps (contoured at 1σ) for monomer A (unliganded) and monomer B (pppGpp-bound), respectively. **(C, D)** Polder OMIT maps (contoured at 3σ) for residues Arg44 and Asp78 in monomer A and monomer B, respectively, confirming the conformational changes induced by pppGpp binding. In monomer A (without pppGpp), Arg44 forms a salt bridge with Asp78; upon pppGpp binding in monomer B, the salt bridge is disrupted, and Arg44

rearranges to coordinate the ligand pppGpp, while Asp78 is released to approach the Mn^{2+} ion.

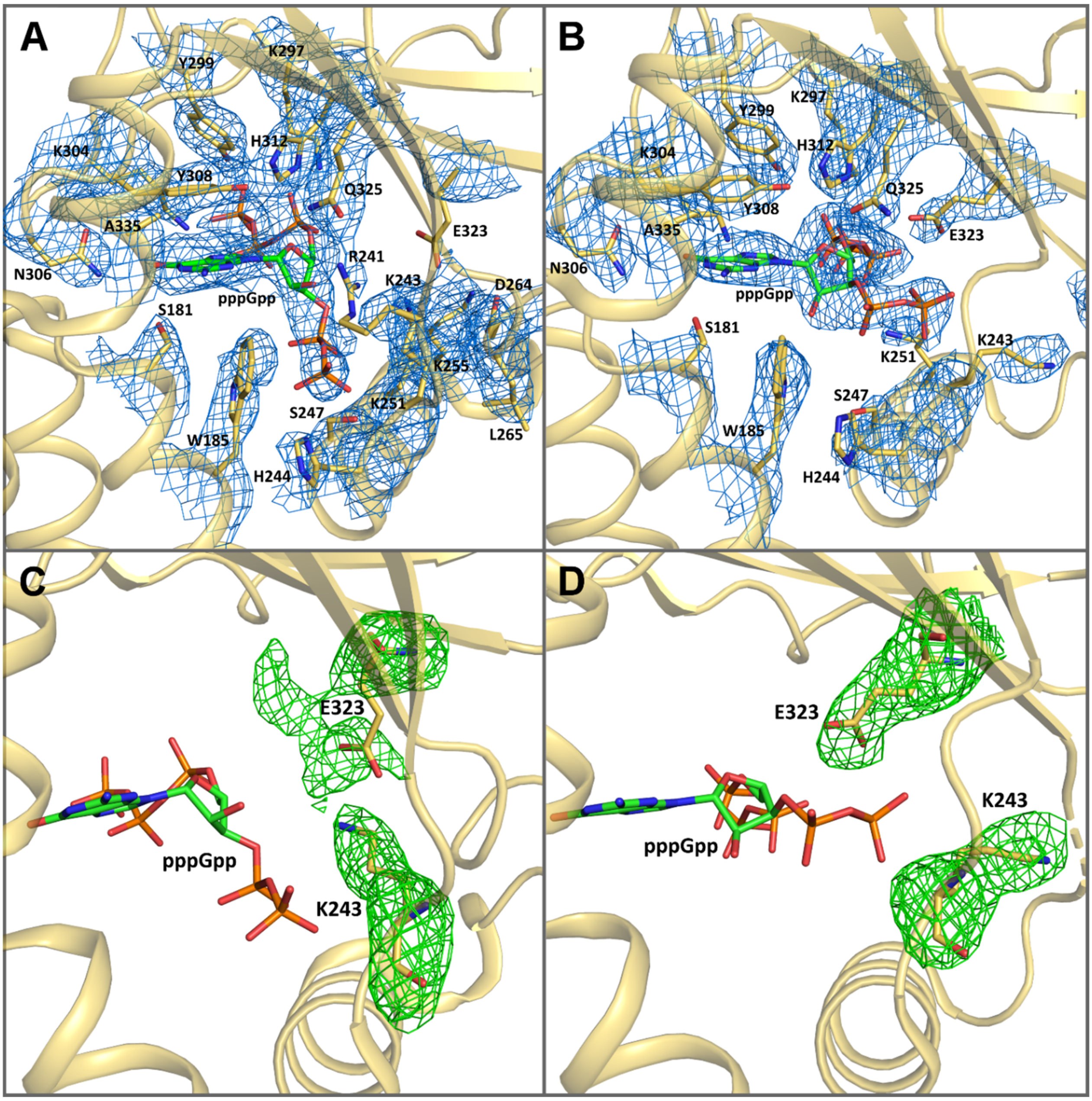

Figure S4. Electron density maps for the Rel_{Seq385} synthetase active site. **(A, B)** 2mFo-DFc maps (contoured at 1σ) for monomer A and monomer B, respectively. **(C, D)** Polder OMIT maps (contoured at 3σ) for catalytically essential residues Lys243 and Glu323 in monomer A and monomer B, respectively. In monomer A, the Glu323 side chain shows poor density on the original maps, consistent with flexibility, as confirmed by the Polder map. In monomer B, Glu323 becomes ordered, while Lys243 rearranges away from the active site.

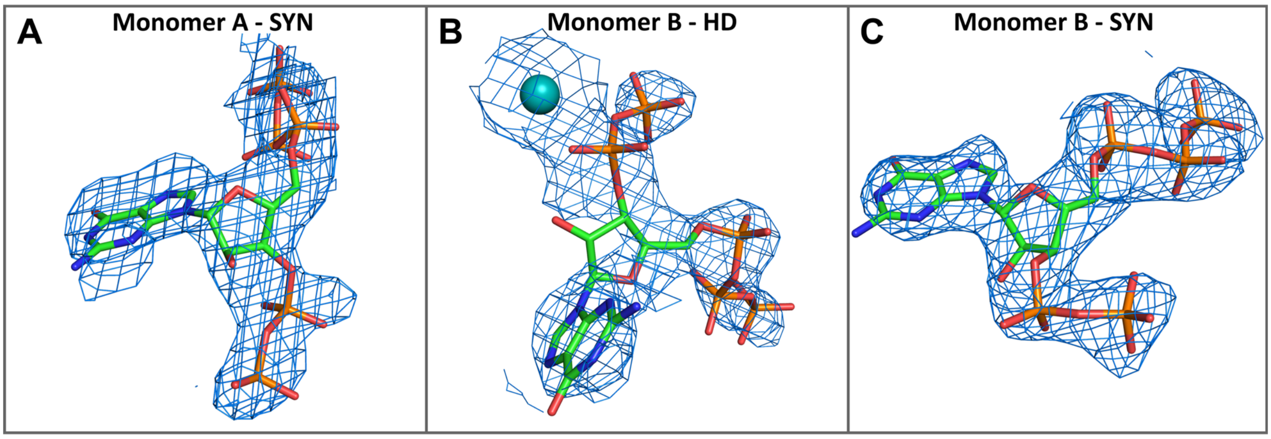

Figure S5. 2mFo–DFc electron density maps (contoured at 1σ) showing pppGpp bound in the synthetase site of monomer A (A), and in the hydrolase (B) and synthetase (C) sites of monomer B.

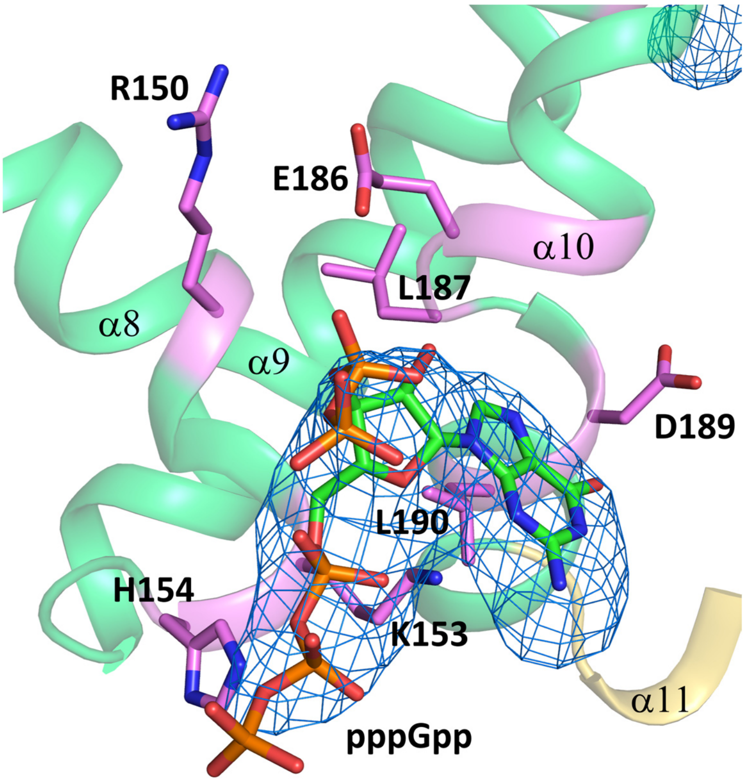

Figure S6. Close-up view of the additional pppGpp binding hotspot in the linker region identified by MD simulations. Residues forming this hotspot are shown as violet sticks and labeled. pppGpp is shown as sticks and color-coded with carbon (green), oxygen (red), nitrogen (blue), and phosphorus (orange).

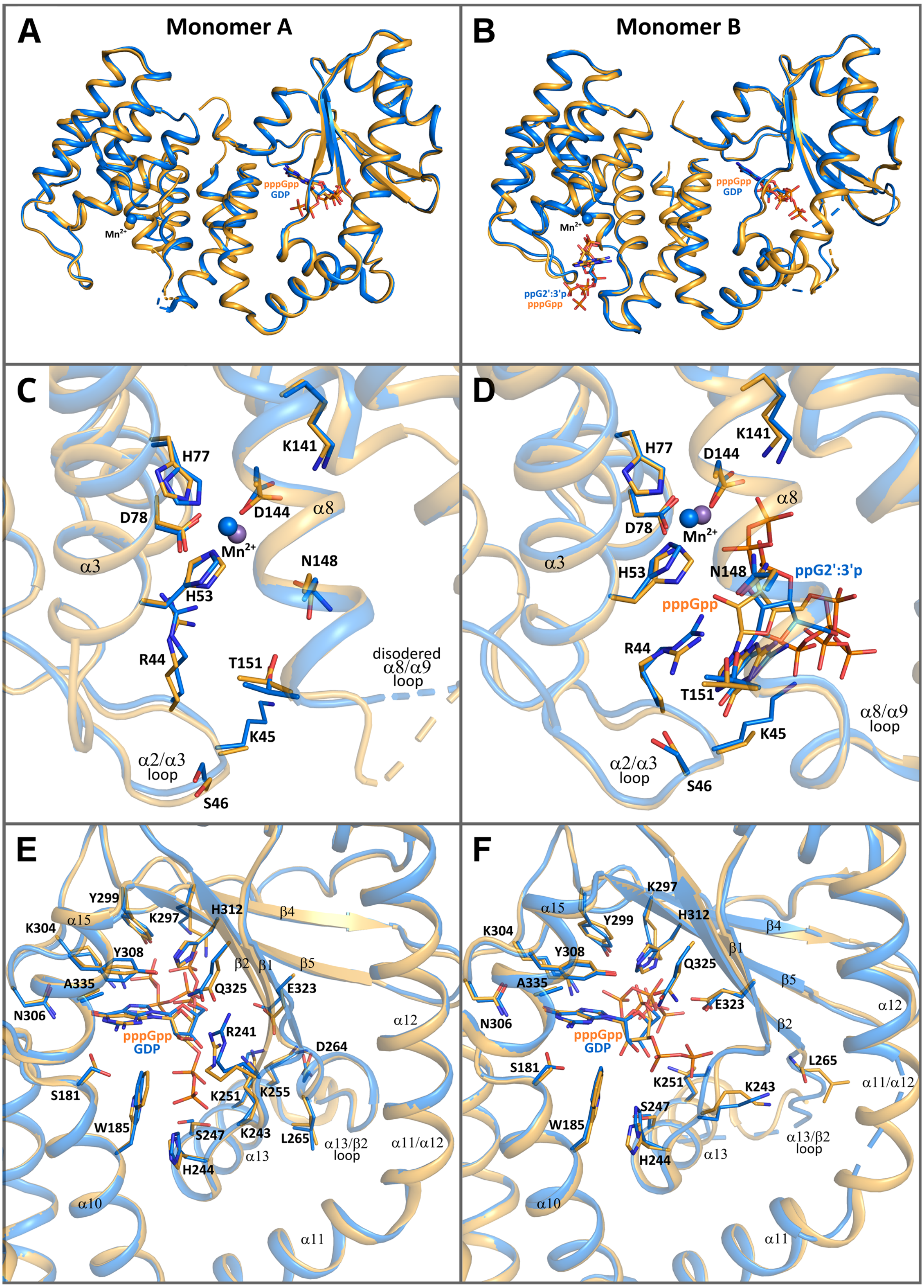

Figure S7. Superposition of Rel_{Seq385} and Rel_{Seq}·GDP structure (PDB: 1VJ7). **(A, B)** Cartoon representation of superimposed Rel_{Seq385} (orange) and Rel_{Seq}·GDP (blue) for monomer A **(A)** and monomer B **(B)**. **(C, D)** Close-up views of the hydrolase (HD) active sites: monomer A **(C)** and monomer B **(D)**.

(E, F) Close-up views of the synthetase (SYN) active sites: monomer A **(E)** and monomer B **(F).** Key catalytic amino acid residues are shown as sticks. Ligands (pppGpp, ppG2':3'p, GDP) are shown as sticks colored according to the monomer color.

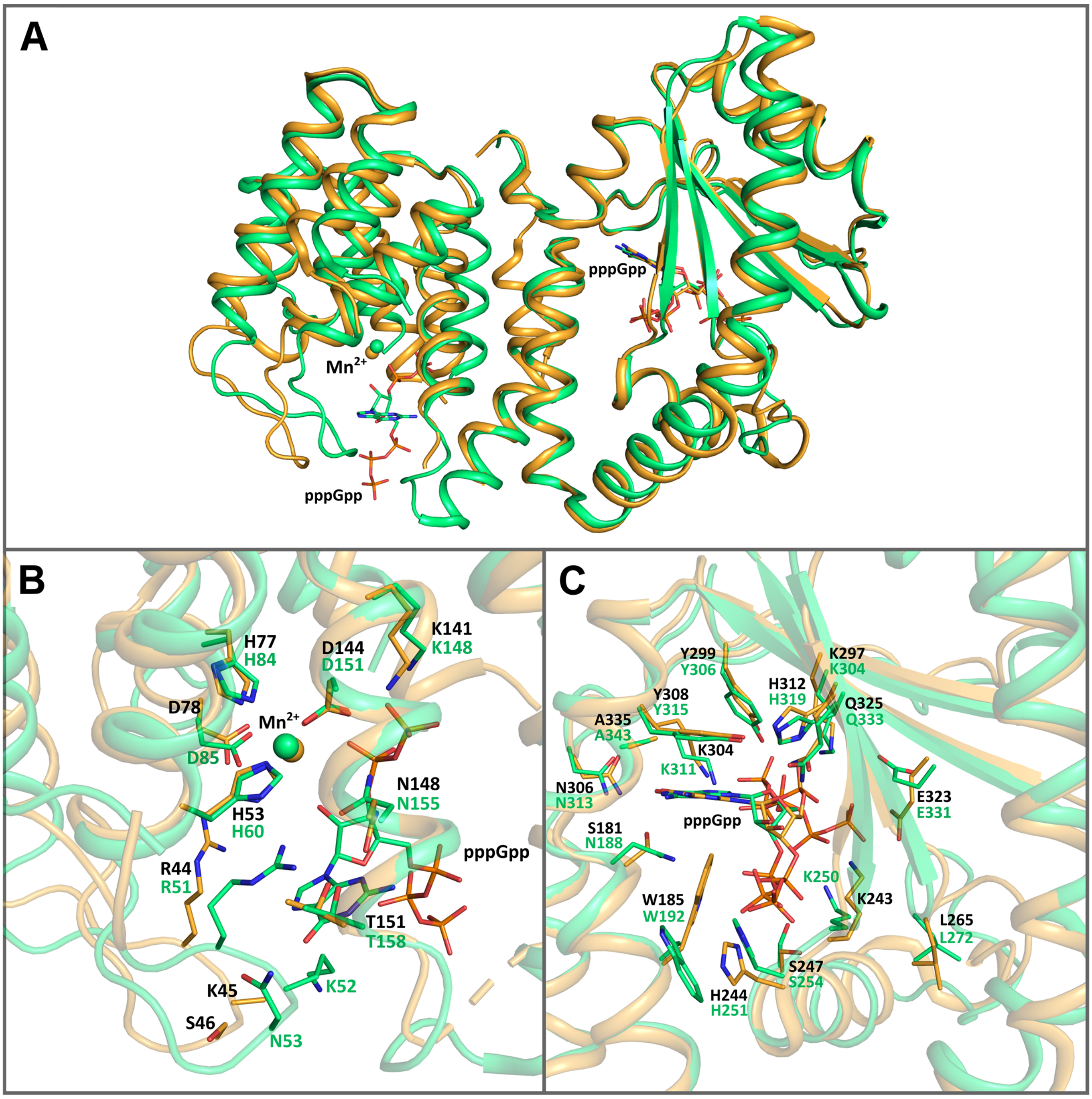

Figure S8. Superposition of the Rel_{Seq385} (**monomer A**) and *S. aureus* Rel (PDB 7OIW, chain B). **(A)** A cartoon representation of superimposed Rel_{Seq385} (orange) and 7OIW (green). The structures were superimposed using all Cα atoms. pppGpp molecules bound in both HD and SYN sites of Rel_{Seq385} and 7OIW are shown as sticks. **(B, C)** Structural alignments of the HD **(B)** and SYN **(C)** active sites, showing key catalytic residues as sticks (labeled for Rel_{Seq385} in black and for 7OIW in green).

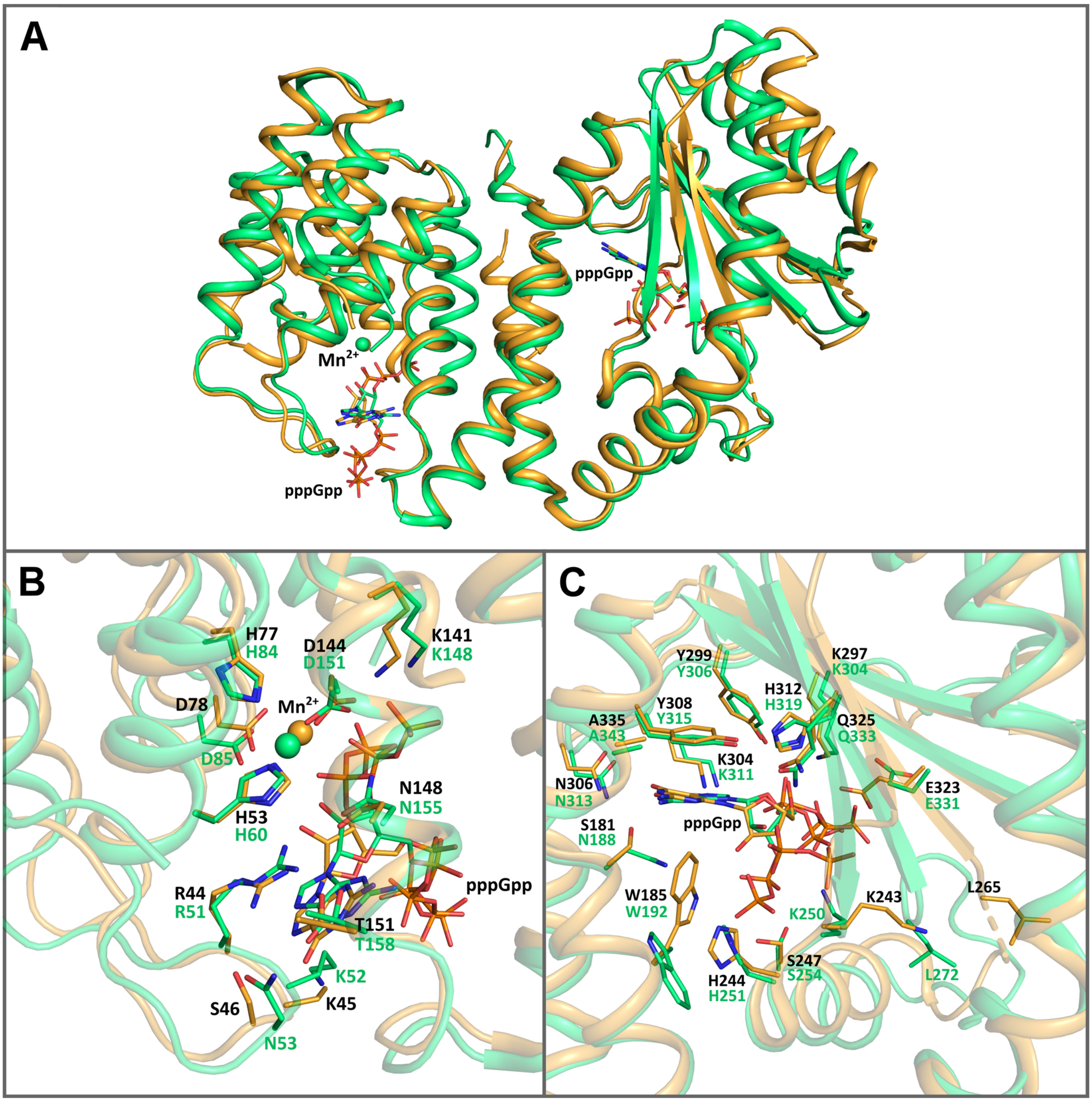

Figure S9. Superposition of the Rel$_{Seq385}$ (**monomer B**) and *S. aureus* Rel (PDB 7OIW, chain B). **(A)** A cartoon representation of superimposed Rel$_{Seq385}$ (orange) and 7OIW (green). The structures were superimposed using all Cα atoms. pppGpp molecules bound in both HD and SYN sites of Rel$_{Seq385}$ and 7OIW are shown as sticks. **(B, C)** Structural alignments of the HD **(B)** and SYN **(C)** active sites, showing key catalytic residues as sticks (labeled for Rel$_{Seq385}$ in black and for 7OIW in green).

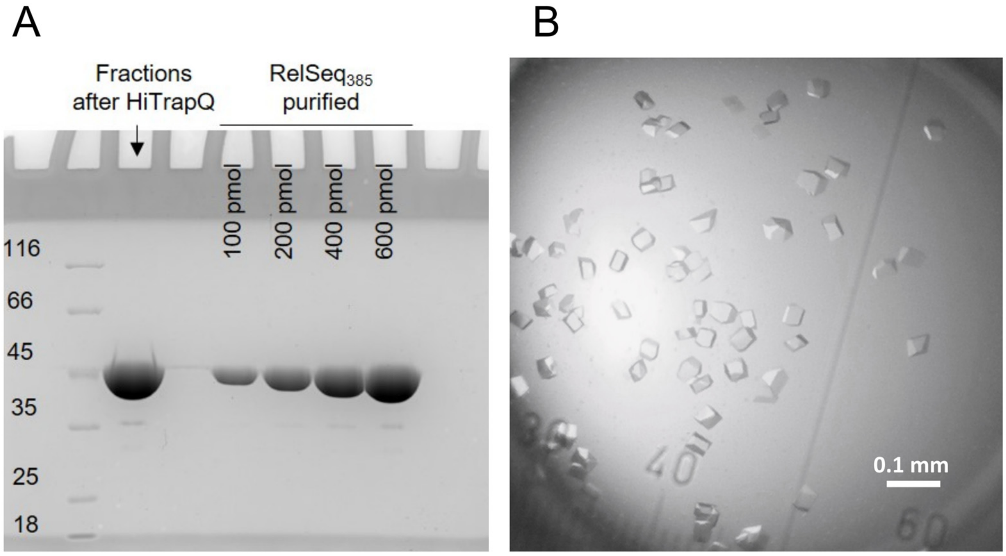

Figure S10. Analysis of Rel$_{Seq385}$ protein. **(A)** 10% SDS–PAGE analysis of purified Rel$_{Seq385}$. (B) Protein crystals of Rel$_{Seq385}$·pppGpp obtained by the hanging-drop method.

Table S1. X-ray data collection and refinement statistics. Values in parentheses refer to the highest resolution shell.

**PDB code**	**24IO**
Wavelength, Å	1.54
Space group	C 2
a, b, c (Å)	173.75, 44.98, 126.43
α, β, γ (°)	90, 110, 90
Resolution range (Å)	118.8 – 3.2 (3.3 – 3.2)
No. of total reflections	48610 (2432)
No. of unique reflections	14251 (713)
Completeness, %	90.9 (49.1)
Multiplicity	3.4 (3.4)
Mean $I/\sigma(I)$	4.2 (1.4)
R_{merge}	0.384 (0.771)
R_{meas}	0.454 (0.912)
R_{pim}	0.239 (0.479)
Half-set correlation $CC_{1/2}$	0.838 (0.522)
Resolution range in refinement, Å	58.52 – 3.20 (3.44 – 3.20)
Reflections in refinement	14180 (796)
Reflections in free set	695 (39)
R_{work}	0.242
R_{free}	0.299
Number of non-hydrogen atoms	5519
macromolecules	5206
ligands	213
solvent	100
Protein residues	657
Average B-factor (Å^2)	43
Wilson B-factor (Å^2)	17
r.m.s. bonds (Å)	0.005
r.m.s. angles (°)	0.795
Ramachandran:<br>favored (%)<br>allowed (%)<br>outliers (%)	 96.41<br>3.43<br>0.16
Rotamer outliers (%)	0.0
Clashscore	7.13
Molprobity score	1.63
